# Circadian clock gene Clock-Bmal1 regulates cellular senescence in Chronic obstructive pulmonary disease

**DOI:** 10.1186/s12890-022-02237-y

**Published:** 2022-11-22

**Authors:** Lingling Li, Min Zhang, Chunyang Zhao, Yusheng Cheng, Chuanmei Liu, Minhua Shi

**Affiliations:** 1 Department of Pulmonary and Critical Care Medicine, The Second Affiliated Hospital of Soochow University, Suzhou, China; 2 Department of Pulmonary and Critical Care Medicine, Yijishan Hospital, Wannan Medical College, Wuhu, China; 3 Department of Emergency, Yijishan Hospital, Wannan Medical College, Wuhu, China

**Keywords:** Circadian clock, Chronic obstructive Pulmonary Disease (COPD), Smoke, Senescence

## Abstract

Chronic obstructive pulmonary disease (COPD) is a progressive respiratory disease. COPD is associated with accelerated lung aging. Circadian clock is believed to play important roles in COPD. Although the circadian molecular clock regulates cellular senescence, there is no information available regarding the impact of COPD. The aim of this study is to investigate the role of the circadian clock protein BMAL1 and CLOCK in cellular senescence in order to understand the cellular mechanisms of accelerated aging of COPD. Bmal1 and Clock levels were assessed in the plasma samples of non-smokers, smokers, and patients with COPD. The regulation of ciracadian clock expression and cell senescence by cigarette smoke extract (CSE) was studied in vitro, and small interfering RNA (siRNA) and overexpression of Bmal1 or Clock were employed to investigate the role of circadian clock on cell senescence. Herein, patients with COPD showed lower Bmal1 and Clock expression in the plasma. Interestingly, CSE exposure contributed to the increased cell senescence, decreased Clock and Bmal1 in human bronchial epithelial cells (Beas-2B cells). We found that knockdown of Clock or Bmal1 lead to upregulation of cell senescence in Beas-2B cells, while overexpression of Clock or Bmal1 inhibited cell senescence in Beas-2B cells, which is through the MAPK pathways. Therefore, our findings indicated that Bmal1 or Clock deficiency may be a significant factor to increase cellular senescence of the lung to develop COPD.

**Supplementary Information:**

The online version contains supplementary material available at 10.1186/s12890-022-02237-y.

## Introduction

Chronic obstructive pulmonary disease (COPD) is a progressive respiratory disease and is considered to be the most important respiratory disease worldwide [1]. Humans will live longer by 2050, 21% of the world’s population will be 60 years or older and the incidence of age-related chronic diseases is also increasing, such as COPD [2–6]. The incidence of COPD was five-fold higher in people aged 65 years than in people aged less than 40 years [7]. The pathogenesis of COPD is still remain unclear, and the main treatment method is to improve the symptoms of COPD patients, but it cannot delay disease progression and reduce mortality. Therefore, new perceptions into the pathogenesis of COPD are required.

The biological clock is a 24-hour internal timekeeping system that affects the rhythms of body’s behavior, physiology, and gene expression [8]. A central pacemaker located in the anterior suprachiasmatic nucleus (SCN) of the hypothalamus, controls the rhythms of behavior and physiology and synchronizes internal time with the external environment in mammals [9]. In addition to the central clock, peripheral tissues (lung, liver and heart) also contain autonomous circadian oscillators that regulate cellular functions and responses to environmental exposure [10–12]. The expression of biological clock genes are regulated by two transcription factors, the positive feedback loops including BMAL1 and CLOCK, and the negative feedback loops including PER and CRY. Activation of the BMAL1/CLOCK transcriptional complex induces the protein level of CRY and PER, which in turn inhibit their own transcription by interacting with BMAL1/CLOCK heterodimers. Circadian clock disruption was found in chronic lung disease, such as Obstructive sleep apnea (OSA) and COPD. This process might be regulated by the subunit α of hypoxia-inducible factor (HIF-1), as its increased level is associated with circadian clock overexpression in OSA [13]. In patients with acute exacerbation of COPD, lung function peaks in the early morning, and FVC, FEV1, and PEF decrease significantly in the night [14, 15]. This may be because cigarette exposure causes changes in circadian clock genes, hormone levels, pulmonary surfactants and mucus hypersecretion, leading to inflammatory responses and a reduction in lung function [16–18].

Cigarette smoking,a major risk factor for COPD, whereas, only 10–15% of smokers develop COPD. This implies there are other factors influence certain individuals. In the process of natural aging, lung function gradually declines and airflow limitation gradually increases. Hence, COPD is a disease associated with accelerated aging [19, 20]. Aging, a gradual decline in homeostasis, leading to an increased incidence of disease [21]. Compared with healthy adults, characteristics of lung aging in COPD patients including declined lung function, enlarged airspace, lossed elasticity and increased cellular senescence. The feature of senescence is morphologic changes, decreased proliferative capacity, and the expression of proteins known as senescence biomarkers [22, 23]. The effects of aging on circadian behavior and metabolism have been clearly documented [24]. Recently, Kondratov et al. have shown that BMAL1^−/−^ mice leads to a significantly shorted lifespan and a premature aging manifestation [25]. Based on the results of Park et al., BMAL1 is associated with telomere length in both zebrafish and mice, and is characterized by rhythmical binding to the ends of chromosomes. [26] However, whether circadian rhythms and aging are associated with COPD and invovled mechanisms remains unclear.

Herein, we explore the role of the core circadian clock protein (BMAL1 and CLOCK) in cellular senescence for clarifying the mechanisms of COPD. In this study, to address this hypothesis, we therefore studied the expression of clock genes in the serum of healthy controls and COPD patients. In addition, we determined the role of circadian clock in cellular senescence in human bronchial epithelial.

## Materials and methods

### Ethics Statement

All patients signed the informed consents. The study protocol was approved by the Medical Ethical Committee of the First Affiliated Hospital of Wannan Medical College. All experiments were performed under the guidelines.

## Human blood specimens

Seventy-six blood samples (30 non-smokers, 20 smokers, and 26 smokers with COPD) were obtained from the First Affiliated Hospital of Wannan Medical College. The plasma was isolated from blood samples and stored at -80 ℃ until further analysis.

## Cell culture

Human bronchial epithelium (Beas-2B) cells were purchased from the Chinese Academy of Sciences on Type Culture Collection Cell Bank (Shanghai, China). Cells were cultured in Dulbecco’s modified Eagle’s medium (DMEM; Gibco) supplemented with 10% fetal bovine serum and 1% streptomycin/penicillin/glutamate solution (Gibco) at 37 ℃ in 5% CO2.

## Assessment of Senescence-associated β-Galactosidase staining

Beas-2B cells were seeded in a 6-well plate with 2 × 10^3^cell/cm^2^. Cells were cultured after 2 days, discard the medium, rinsed cells once with PBS, add 1 ml of fixative to each well for 15 min and then rinsed three times with PBS. Then 1 ml per well of working solution of β-galactosidase with X-Gal was placed, and the plate was maintained at 37^°^C overnight (senescence-associated β-galactosidase staining kit from Beyotime, China). The cells were observed under an inverted microscope.

## Preparation of Aqueous cigarette smoke extract

CSE was prepared using a modification to a previously modified method [27–29]. The total particulate matter (TPM) content of Marlboro Red cigarette was 10 mg/cigarette, tar (10 mg/cigarette), and nicotine (0.8 mg/cigarette). Preparing fresh CSE for each experiment before use.

## Transfection with siRNA

Three sequences for Clock and Bmal1 siRNAs (GenePharma, Shanghai, China) was designed and a control siRNA.The siRNAs were transfected into Beas-2B cells using Lipofectamine 3000 (invitrogen) reagent for 48 h according to instructions.

## Plasmids

Plasmids for expressing CLOCK (pcDNA-CLOCK) or BMAL1 (pcDNA-BMAL1) were purchased from GenePharma (Shanghai, China). For overexpression assays, 2.5 µg plasmids were transfected with Lipofectamine 3000 (invitrogen) reagent into the cells according to the reagent instructions.

## Western blot analysis

Total cell protein was extracted by PMSF RIPA lysis method. Samples were separated by SDS/PAGE (Invitrogen, USA) and then transferred to nitrocellulose (NC) membranes. Membranes were blocked with 5% BSA for 1 h and then incubated with the indicated primary antibody overnight at 4 °C. Antibodies against: Clock (Cat# ab93804, Abcam), Bmal1 (Cat# ab93806, Abcam), pH2AX (Cat# ab81299, Abcam), phospho-ERK (Cat# 4370, CST), phospho-P38 MAPK (Cat# 4511, CST), ERK (Cat# 4695, CST), P38 MAPK (Cat# 8690, CST), and GAPDH (Cat# ab8245, Abcam). The gray values of protein bands were measured using Image Lab 2.0 software (Bio-Rad). GAPDH was used as the loading control.

## RNA extraction, reverse transcription and real-time RT-PCR

Total RNA was isolated following the user’s manual using trizol reagent (Invitrogen). Quantitative PCRs were performed using the SYBR Green (Takara). The primers are provided in supplemental Table 1.

### ELISA

Enzyme-linked Immune Sorbent Assay kit was used to assess serum protein concentrations: BMAL1and CLOCK (EIAab, Wuhan, China). BMAL1 and CLOCK were detected using ELISA kits according to the manufacturers’ instructions.

## Statistical analyses

Statistical analyses were carried out with SPSS 25.0 software and GraphPad Prism 6.0. Normality of data distribution was assessed prior to the application of parametric tests. The results are shown as the mean ± SD. Data were analyzed by one-way analysis of variance between multiple groups and t test between two groups. The level of significance was set at *p* < 0.05.

## Results

### Decreased clock and Bmal1 level in the serum of COPD patients

**Fig. 1:**
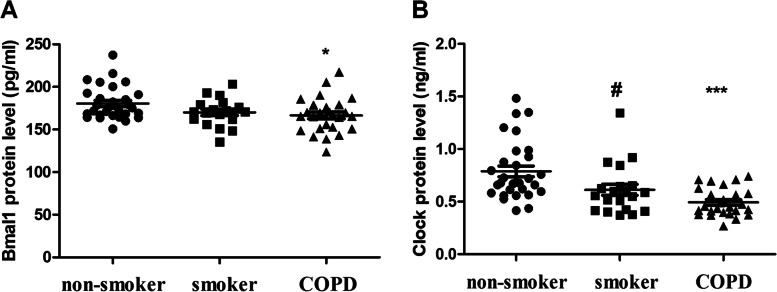
Decreased Bmal1 and Clock protein level were observed in the plasma of COPD patients ** A**.Plasma were collected from non-smokers (*n* = 30), smokers (*n* = 20), and COPD (*n* = 26). **A** Bmal1 protein level. **B** Clock protein level. #*p* < 0.05, *p < 0.05, ***p < 0.001 vs. non-smokers

Based on the patients’ lung function and smoking history, we divided the 76 subjects into three groups: non-smokers, smokers and smokers with COPD group. We summarizes the clinical characteristics of the subjects in Supplementary Table 2. The three groups were similar in terms of age and body mass index. The levels of Clock and Bmal1 in the serum of COPD patients were significantly lower than those of healthy controls (Fig. 1 A and B). As previously reported, disturbances in the circadian rhythm are involved in the development of COPD.

## Cigarette smoke down-regulate Bmal1 and clock expression in bronchial epithelial cells

**Fig. 2:**
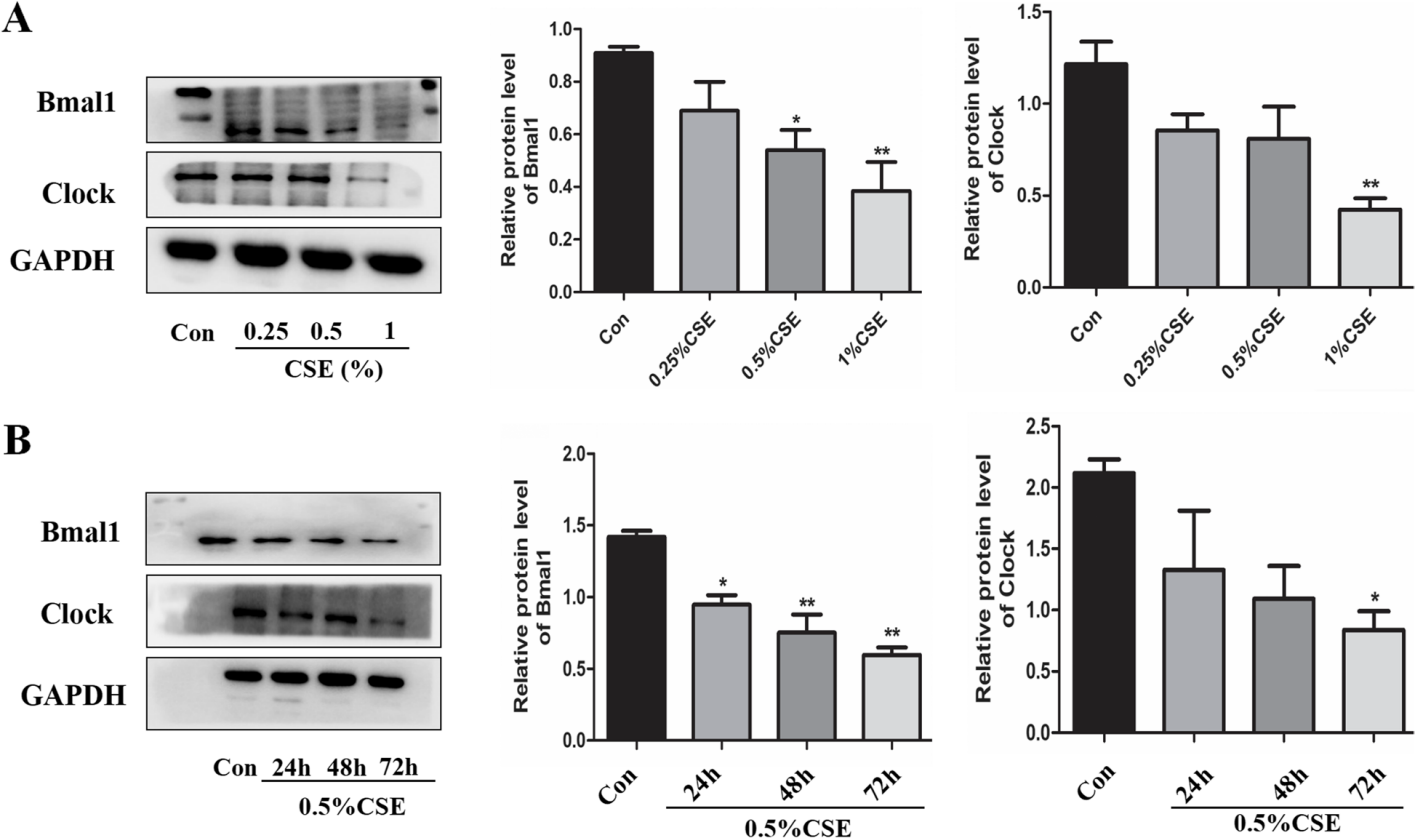
CSE decreased the expression of Bmal1 and Clock in Beas-2B cells

Following a different concentration of CSE stimulated, the expression of Bmal1 and Clock were decreased significantly at concentration of 1%CSE (Fig. 2A). To explore the association between clock genes and CSE in COPD, we also treated Beas-2B cells with 0.5%CSE for 24 h, 48 and 72 h. We observed a decreased Bmal1 and Clock in Beas-2B cells after 72 h of CSE (Fig. 2B).

## Higher levels of cellular senescence in CSE-induced bronchial epithelial cells

**Fig. 3:**
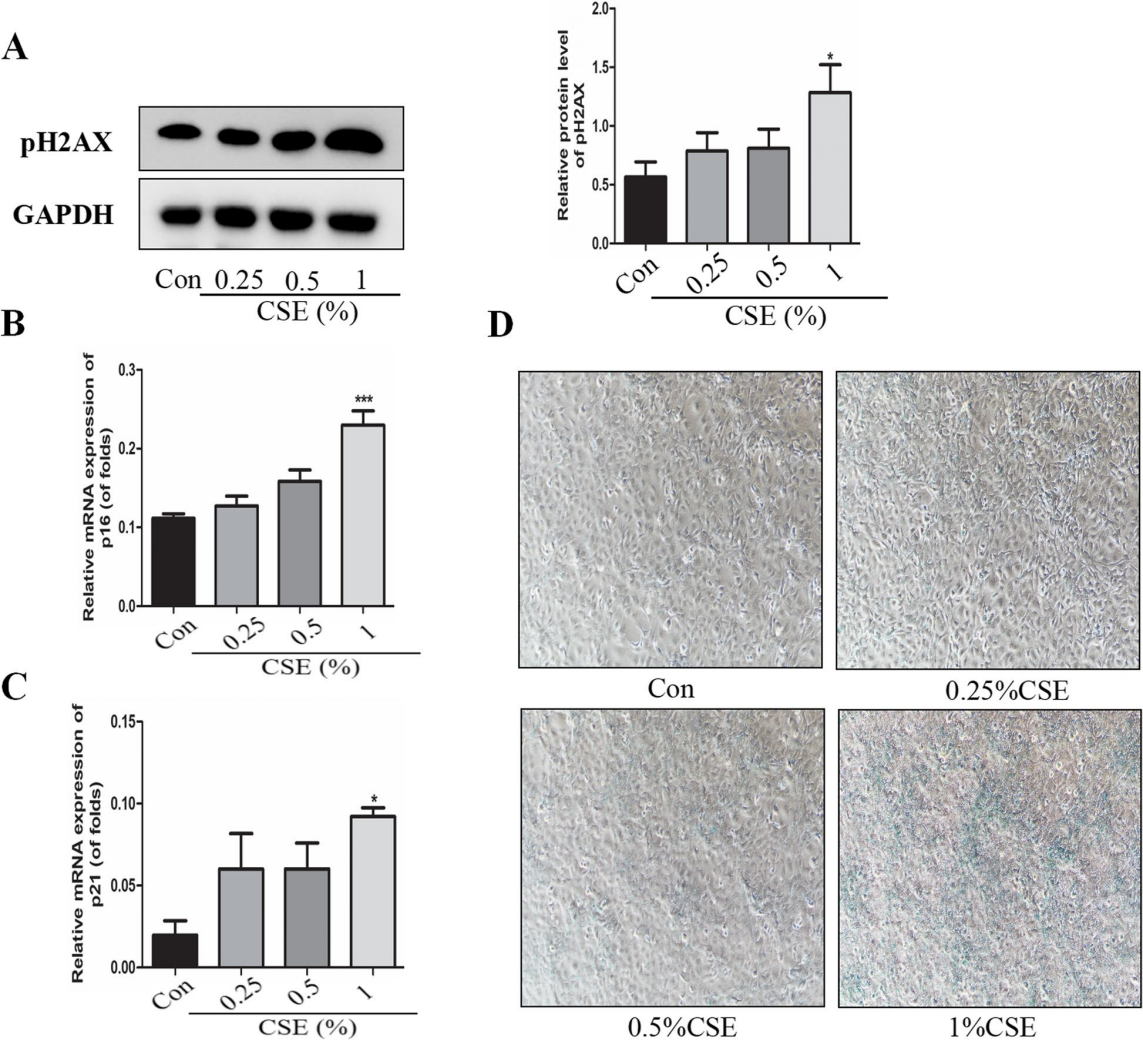
CSE increased cellular senescence in Beas-2B cells

Cigarette Smoke leads to accelerated aging in Beas-2B cells. CSE (0.25%, 0.5% and 1%) stimulated Beas-2B cells for 24 h, respectively. As shown in Fig. 3A, phosphorylated H2AX (pH2AX) was increased in a concentration-dependent manner. Phosphorylated H2AX is a marker of cellular senescence and DNA double-strand breaks (DSB) [30, 31]. And the mRNA expression of p16 and p21 were also increased after 24 h of CSE (Fig. 3B and C). Cells were seeded in six-well plates and then stimulated with CSE (0.25%, 0.5% and 1%) for 24 h and cellular senescence was detected the next day. An increase of cell senescence was showed by the senescence associated-gal staining analysis (Fig. 3D).

## Cigarette smoke triggered circadian disturbance in bronchial epithelial cells

**Fig. 4:**
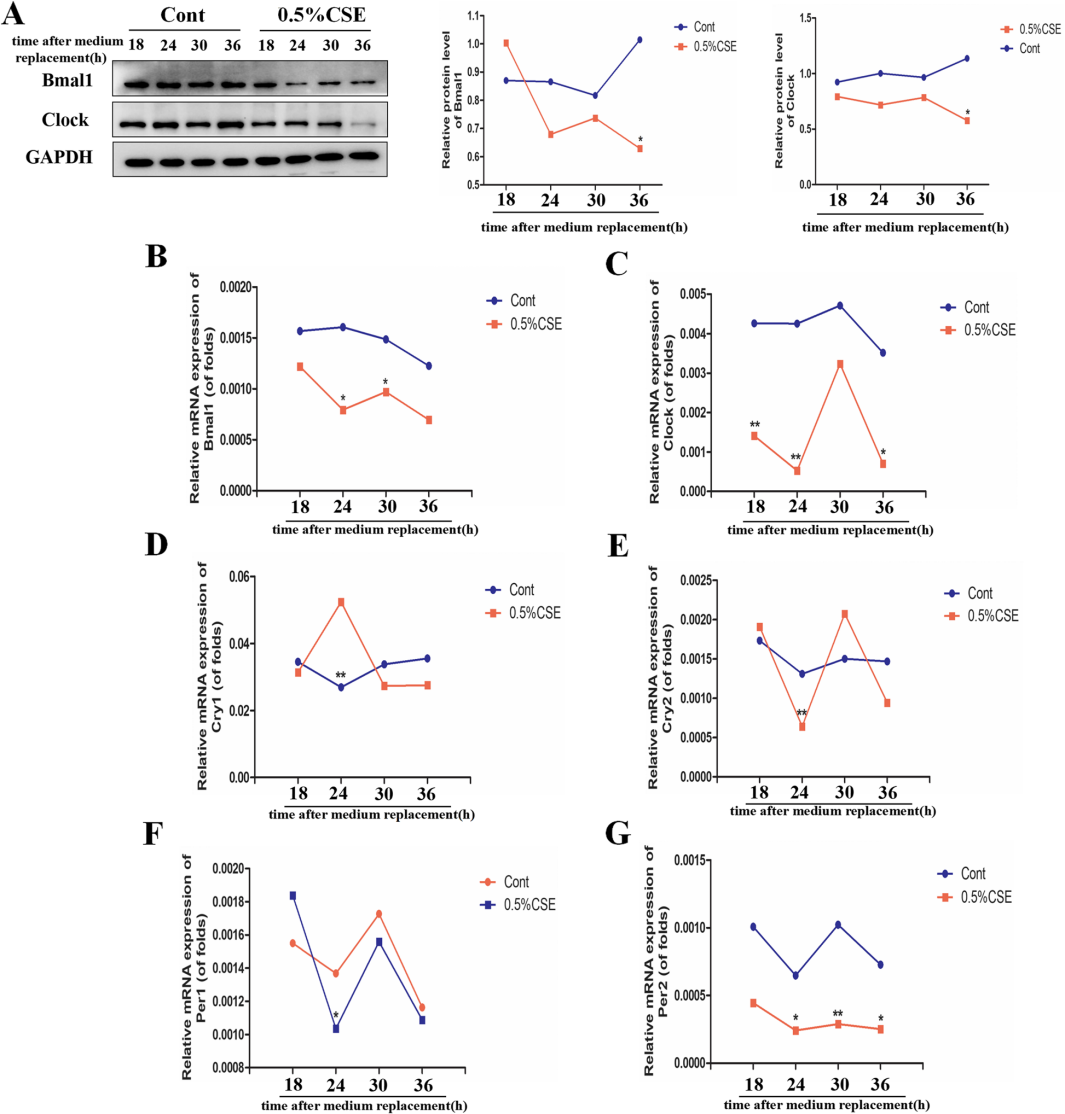
CSE induced circadian disturbance Beas-2B cells

To further investigate the effect of CSE on the circadian rhythms in COPD, we stimulated Beas-2B cells with CSE for 24 h. In our study, we found Bmal1 and Clock showed a regular rhythmic expression in the control group in 24 h cycle. However, 0.5%CSE significantly disturbed the circadian rhythms (Fig. 4A). Then, we detected the mRNA expression of other core clock genes (Fig. 4B-G). We observed that CSE disturbed the mRNA expression of Clock, Bmal1, Per1, Per2, Cry1 and Cry2. We also found that the expression levels of Bmal1 and Clock are lowest at Zeitgeber time 24 (ZT24), nevertheless Cry1 peaked at ZT24. Cry1 mRNA expression seemed to be inversely correlated with Bmal1 and Clock mRNA level.

## Knockdown of Bmal1 and clock increased cell senescence in bronchial epithelial cells

**Fig. 5:**
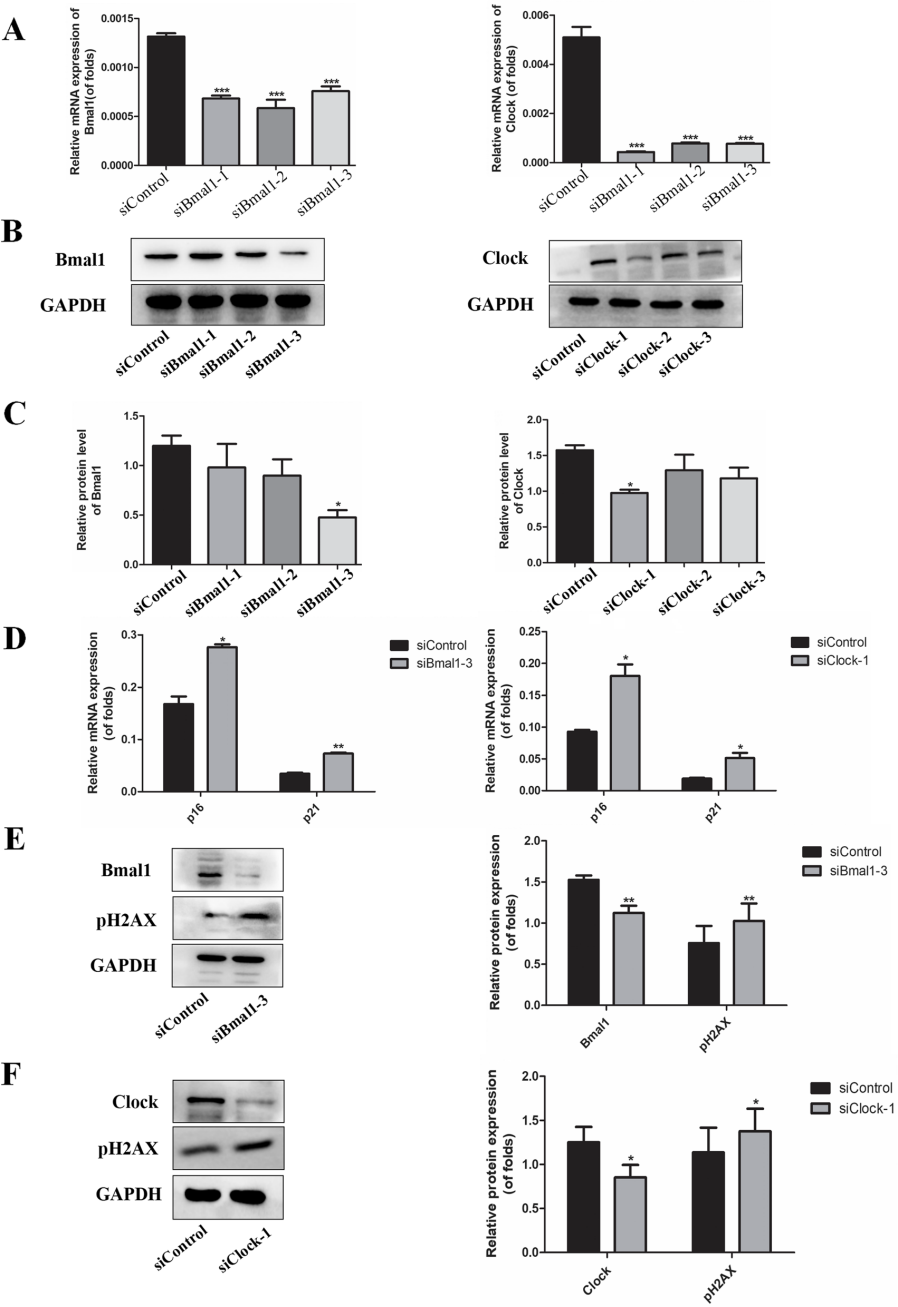
Knockdown of Bmal1 or Clock stimulated cellular senescence in Beas-2B cells

To study the association between circadian rhythms and cellular senescence, Beas-2B cells were transfected with Bmal1 siRNA (siBmal1-1, siBmal1-2, and siBmal1-3) or Clock siRNA (siClock-1, siClock-2, and siClock-3) for 48 h, and then mRNA and protein expression was detected. The cells transfected with Bmal1 siRNA and Clock siRNA showed a lower expression of two genes (Fig. 5 A-C). Based on the results, we used siBmal1-3 and siClock-1 in the subsequent experiments. After knocking down Bmal1 and Clock, we found that the mRNA levels of p16 and p21 were significantly increased (Fig. 5D). Besides, phosphorylated H2AX was augmented significantly in Bmal1-KD cells and Clock-KD cells (Fig. 5E and F). The results suggest that the depletion of circadian clock accelerated aging in bronchial epithelial cells.

## Overexpression of Bmal1 and clock repressed cell senescence in bronchial epithelial cells

To further examing the effect of Bmal1 and Clock on cell senescence in bronchial epithelial cells, cells overexpressing.

**Fig. 6:**
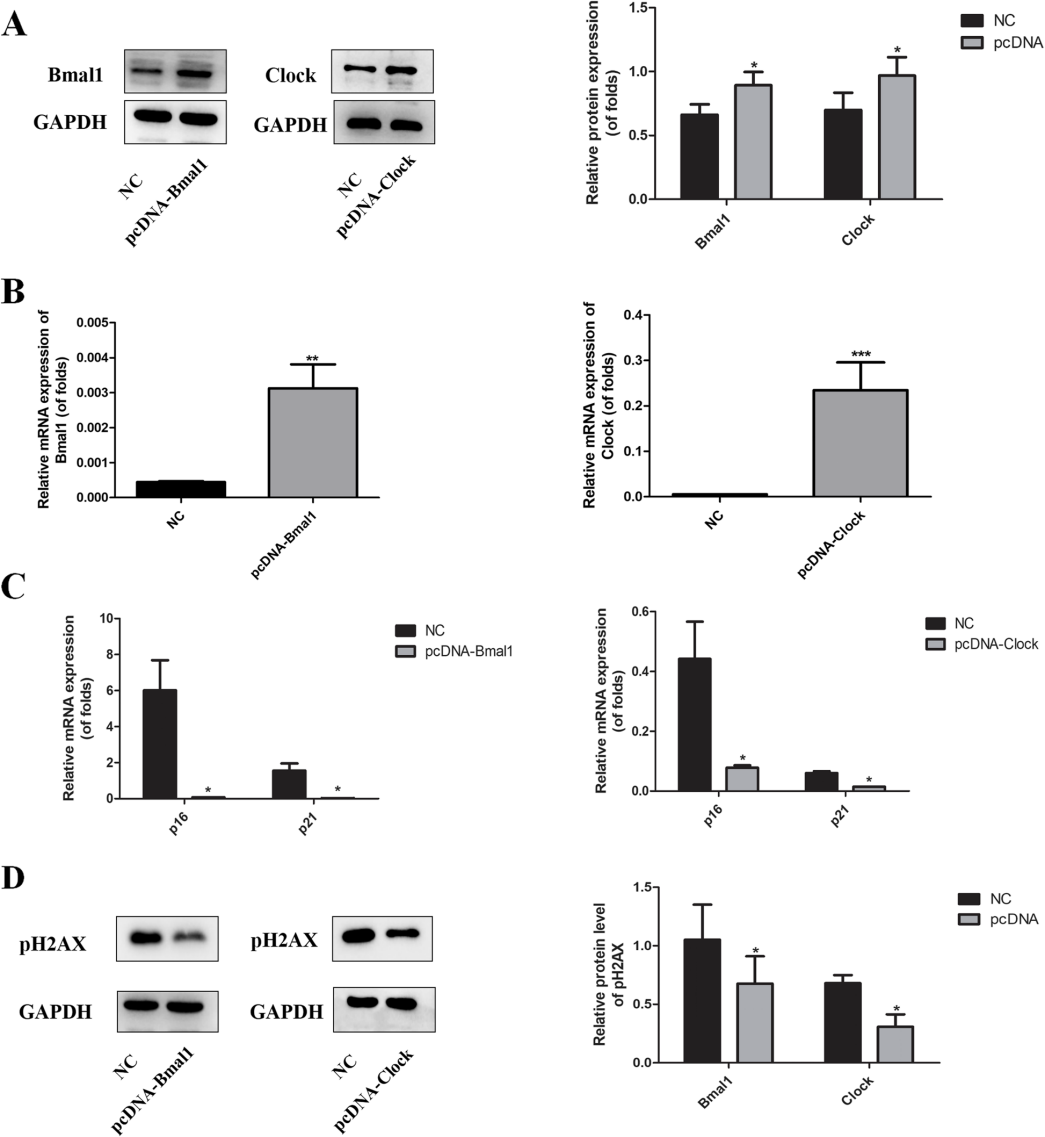
Overexpression of Bmal1 or Clock inhibited cellular senescence in Beas-2B cells

Bmal1 and Clock were established. Western blot and RT-PCR analyses confirmed the increase in Bmal1 and Clock protein expression, respectively (Fig. 6 A and B). As seen in Fig. 6 C, the mRNA levels of p16 and p21 were significantly repressed. Western blot analyses showed that overexpression of Bmal1 and Clock inhibited phosphorylated H2AX (Fig. 6D).

## Dexamethasone influence the expression of Bmal1 and clock in bronchial epithelial cells

**Fig. 7:**
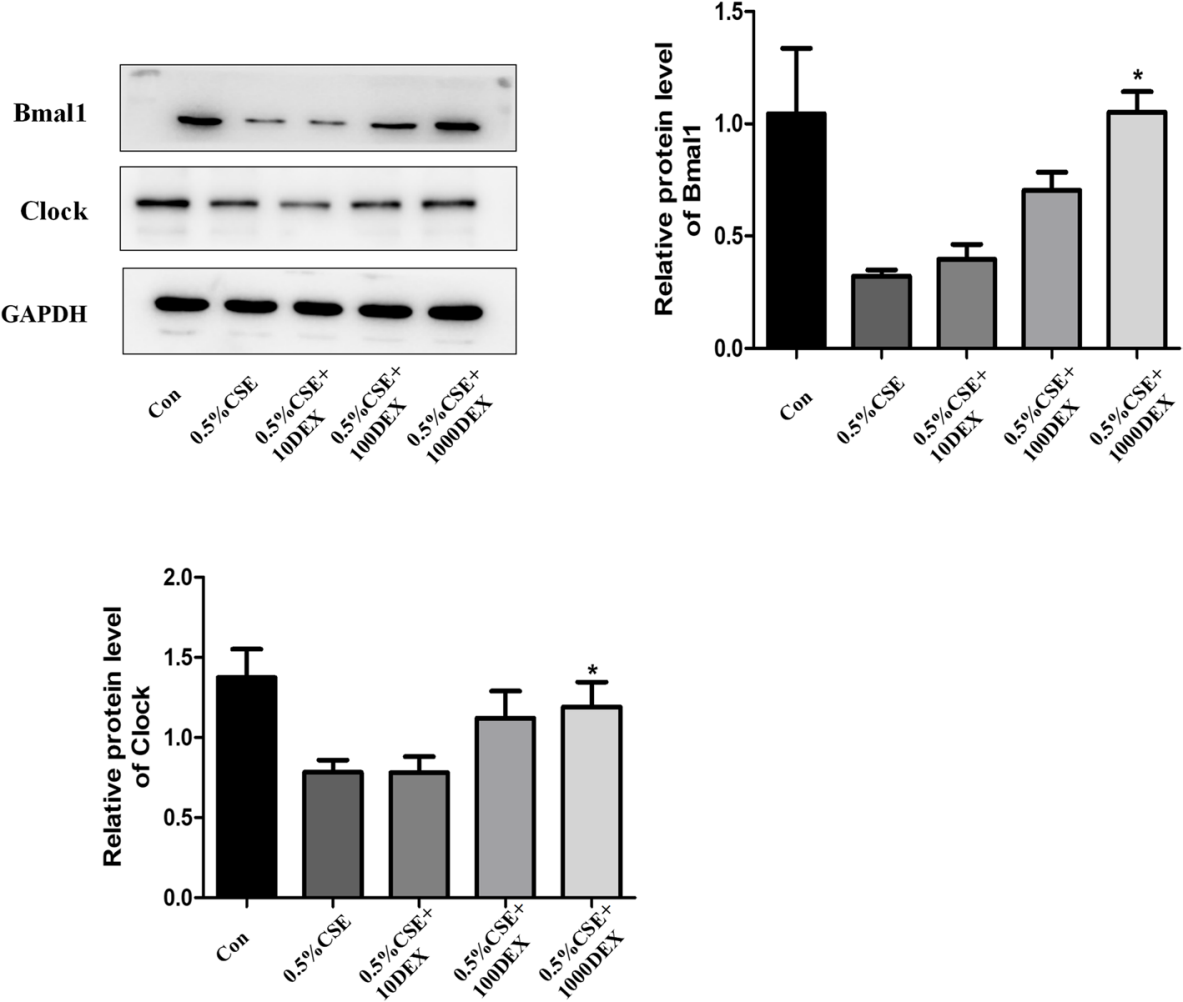
Effect of dexamethasone on the protein expression of Bmal1 and Clock in Beas-2B cells

Dexamethasone (DEX), an agonist of the glucocorticoid receptor, is well known to reset cellular clocks [32]. Cells were pretreated with Dex (10, 100, and 1000 pM)for 1 h prior to incubation with 0.5% CSE for 24 h. We showed that Dex increased the expression of Bmal1 level in Beas-2B cells stimulated by CSE. In addition, Dex also significantly increased Clock protein level in Beas-2B cells (Fig. 7).

## Circadian clock regulate cell senescence via MAPK pathways in bronchial epithelial cells

**Fig. 8:**
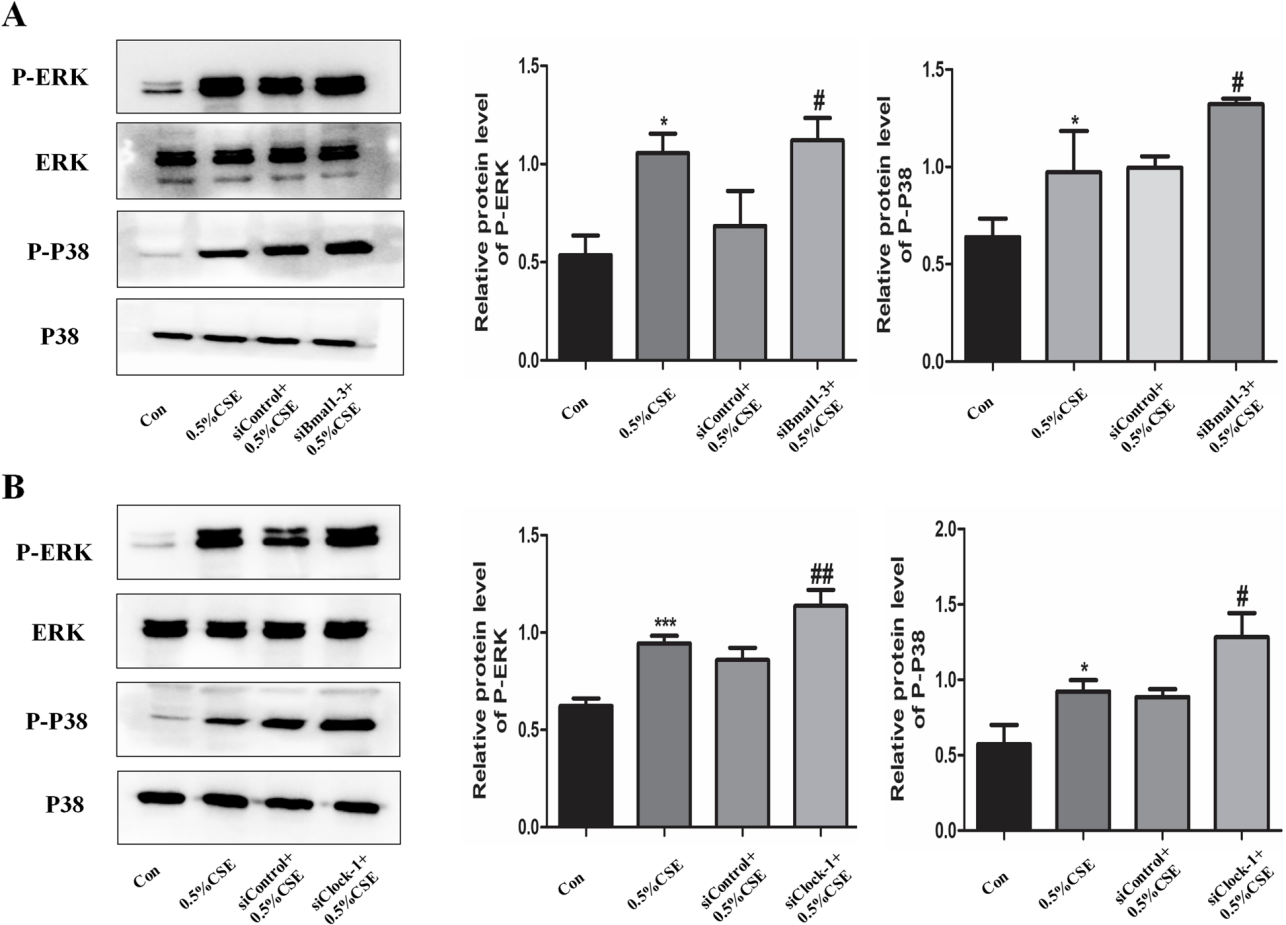
Knockdown of Bmal1 or Clock modulates cigarette smoke-induced cell senescence: role of intracellular signalling pathways

**Fig. 9:**
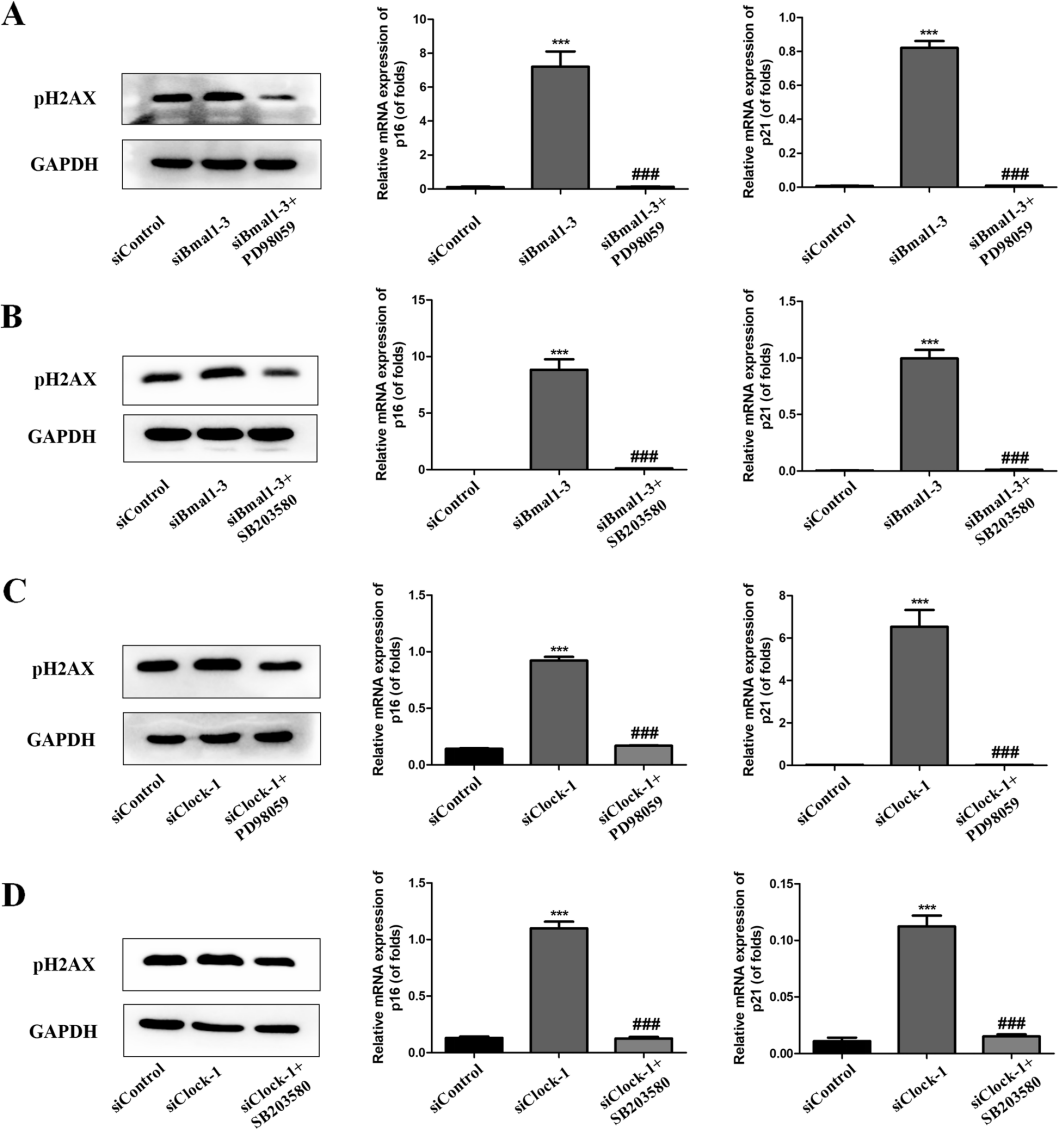
MAPK inhibitors reversed the up-regulation senescence in Bmal1 or Clock knockdown cells

In previous studies, MAPK signaling pathways were closely related to the mechanism of COPD. In the present study, Beas-2B cells were stimulated with 0.5% CSE for 1 h and MAPK pathways were activated by CSE. Furthermore, we also established Bmal1 knockdown cells and Clock knockdown cells, then cells were incubated with 0.5% CSE for 1 h. Our results showed that CSE-induced P38 and ERK phosphorylation was also upregulated after knocking down Bmal1 and Clock (Fig. 8 A and B). To further confirm whether circadian clock regulates cell senescence through MAPK pathways, the pathway inhibitors PD98059 (20 µM) and SB203580 (20 µM) were used. Bmal1 knockdown cells or Clock knockdown cells were pretreated with PD98059 and SB203580 for 1 h. As shown in Fig. 9, the MAPK inhibitors reversed the up-regulated cellular senescence expression after knocking down Bmal1 and Clock. The result indicates that MAPK signalling pathways were involved in the anti-aging functions of circadian clock.

## Discussion

COPD patients exhibit daily rhythms with increased symptoms associated with sleep disruption [33]. Due to the daily nature of COPD exacerbation and its influence on sleep quality, we suggested that disruption of the circadian rhythm may be a vital role in the pathogenesis of COPD. Several pathways by which circadian clock genes control life cycle regulation and organ aging. In this study, we investigated two core clock genes, BMAL1 and CLOCK, in regulating cellular senescence in COPD. We are the first time showing that level of Clock-Bmal1 were reduced in the serum of COPD patients, and CSE caused the inhibition of expression of Bmal1 and Clock coincided with increased expression of cell senescence in human bronchial epithelial cells, which is through the MAPK pathways. The data might uncovered new insights about the accelerated aging of lung the in the development of COPD .

CS exposure changed clock genes expression of the lung in both rats and mice [34, 35]. The expression of p16 and p21 were increased in Emphysematous lungs, which are markers of cell senescence [36]. In our study, We demonstrated that CSE significantly reduced the expression of Bmal1 and Clock. We also found that cell senescence was induced by CSE in Beas-2B cells. This notion is basically the same as that of a previous study, acrolein treatment caused deletion of Clock and Bmal1 in HUVECs [37]. Recently, other studies have shown that disruption of the circadian molecular clock has a significant effect on lung function and lung pathophysiology [38–40]. A recent study also finds link between biological clock and aging [41]. Therefore, we hypothesized that circadian clock might accelerated aging process in COPD.

Disturbance in circadian rhythms are involved in the pathological processes of many diseases, such as inflammatory and metabolic diseases [42]. It has been found sleep disturbances and night-time symptoms are common in COPD patients, suggesting disrupted circadian rhythms in COPD patients [43]. Oishi and colleagues are the first found Clock gene expression in the lungs [44] and Gibbs and colleagues [38] reported molecular clock function in bronchial epithelial cells. We are the first time found that CSE treatment disrupted rhythms of Clock and Bmal1 in bronchial epithelial cells. In addition, we also found the relation between circadian rhythms and cell senescence. The expression of p16, p21 and pH2AX increased significantly in Clock or Bmal1 knockdown cells. However, over-expression of Bmal1 or Clock decreased the expression of p16, p21 and pH2AX significantly. These results are in line with a previous study that Bmal1^−/−^ mice display early signs of aging, possibly via cellular senescence [25]. In the present study, we have found that circadian clock dysfunction contributes to accelerated cellular senescence in bronchial epithelial cells. Therefore, our results showed that circadian clock may accelerated lung cellular senescence and premature aging, the pivotal pathogenesis of COPD.

MAPK pathways play a vital role in the development of COPD. In our study, we demonstrated that circadian clock regulated cellular senescence through the MAPK pathway. In a previous study, Cellular senescence can promote the transformation of benign skin papilloma to cancer by enhancing p38 MAPK and ERK MAPK signaling pathways [45]. Goldsmith et al. found that circadian clock genes were regulated by MAPK pathways in mammalian SCN [46]. Therefore, we speculated that MAPK pathway is likely to be involved in circadian clock regulating CSE-induced cell senescence. In our study, CSE significantly activated p38 MAPK and ERK MAPK pathway, whereas knockdown of Bmal1 or Clock further upregulated the effect of CSE on the MAPK pathways in Beas-2B cells. We further used the p38 and ERK inhibitor (SB203580 and PD98059) to confirm the above results, which reduced the increased cell senescence after knocking down Bmal1 or Clock. Our findings implied that circadian clock regulated cellular senescence in response to CSE.

In our study, DEX increased the protein level of Bmal1 and Clock in Beas-2B cells. Accumulating evidence has confirmed that dexamethasone could induced the expression of clock genes in bronchial epithelial cells, peripheral blood mononuclear cells, lymphocytes, and fibroblasts [47]. These findings suggest that molecular clock genes is mechanistically associated with the anti-inflammatory effects of glucocorticoids. Therefore, designing compounds that normalize the expression of circadian clock genes in the lung may be a new approach to treating COPD in the future. In addition, Molecules that enhance circadian clock genes have been developed as an effective alternative to steroid hormones in the treatment of COPD. However, it remains to be seen whether these chronopharmacological drugs can be used to effectively treat cellular senescence in COPD.

The present study has several weakness. Firstly, this study was conducted only in one cell line, whereas, we should use primary cells from patients and controls ideally. Secondly, the exact mechanism by which CSE alters circadian clock levels is unclear, and further studies are needed to clarify the mechanism of CSE-induced deletion of Bmal1 and Clock, and whether genetic modifications of Bmal1 or Clock can subtle aging processes in COPD models.

In summary, we revealed that Clock-Bmal1 may be a vital factor to accelerate cellular senescence of the lung leading to COPD. The study provides data for circadian clock level in the plasma of COPD patients, and further searches the potential role of circadian clock in the development of COPD in vitro studies, which might shed a light on a novel mechanism linked to the accelerated lung aging in the COPD formation.

B.CSE decreased the protein levels of Bmal1 and Clock in Beas-2B cells at concentrations of CSE (0.25– 1%) for 24 h. Relative densities (% of control) are shown of Bmal1 and Clock in Beas-2B cells at concentrations of CSE (0.25– 1%) for 24 h. B. 0.5% CSE decreased the protein levels of Bmal1 and Clock in Beas-2B cells at times of CSE treatment (24–72 h). Relative densities (% of control) are shown of Bmal1 and Clock in Beas-2B cells at times of CSE (24–72 h). Values are expressed as means ± SD of 3 replications. *p < 0.05, **p < 0.01 vs. control group. Full-length gels are presented in Supplementary Fig. 1.

A.CSE increased the protein levels of pH2AX in Beas-2B cells at concentrations of CSE (0.25– 1%) for 24 h. Relative densities (% of control) are shown of pH2AX in Beas-2B cells at concentrations of CSE (0.25–1%) for 24 h. B. Cultured cells were exposed to CSE (0.25%, 0.5% and 1%) for 24 h, The mRNA expression of p16 was examined by real time PCR. C. Cultured cells were exposed to CSE (0.25%, 0.5% and 1%) for 24 h, The mRNA expression of p21 was examined by real time PCR. D. Cultured cells were exposed to CSE (0.25%, 0.5% and 1%) for 24 h, Senescence-associated β-galactosidase (SA-β-gal) activity was assessed. Values are expressed as means ± SD of 3 replications. *p < 0.05, ***p < 0.001 vs. control group. Full-length gels are presented in Supplementary Fig. 2.

A.Beas-2B cells were incubated with CSE (0.5%) at 6 h intervals between 6 and 24 h time points. The protein levels of Bmal1 and Clock were examined by Western blotting. Relative densities (% of control) are shown of Bmal1 and Clock in Beas-2B cells. B-G. The mRNA expression of clock genes Bmal1, Clock, Cry1, Cry2, Per1 as well as Per2 with GAPDH as an internal reference. Values are expressed as means ± SD of 3 replications. *p < 0.05, **p < 0.01 vs. control group. Full-length gels are presented in Supplementary Fig. 3.

A.Beas-2B cells were transfected with Bmal1 and Clock siRNAs separately. The mRNA expression of Bmal1 and Clock were detected by PCR. B. Beas-2B cells were transfected with Bmal1 and Clock siRNAs separately. The protein expression of Bmal1 and Clock were detected by Western blotting. C. Relative densities (% of control) are shown of Bmal1 and Clock in Beas-2B cells. D. p16 and p21 mRNA expression after knockdown of Bmal1 or Clock. E,F. pH2AX protein expression after knockdown of Bmal1 or Clock. Values are expressed as means ± SD of 3 replications. *p < 0.05, **p < 0.01, ***p < 0.001 vs. control group. Full-length gels are presented in Supplementary Fig. 4.

A.Beas-2B cells overexpressing Bmal1 or Clock were established. The protein expression of Bmal1 and Clock were detected by Western blotting. Relative densities (% of control) are shown of Bmal1 and Clock in Beas-2B cells. B. Beas-2B cells overexpressing Bmal1 or Clock were established. The mRNA expression of Bmal1 and Clock were detected by PCR. C. p16 and p21 expression after overexpression of Bmal1 or Clock. D. pH2AX protein expression after overexpression of Bmal1 or Clock. Values are expressed as means ± SD of 3 replications. *p < 0.05, **p < 0.01, ***p < 0.001 vs. control group. Full-length gels are presented in Supplementary Fig. 5.

Cells were pretreated with dexamethasone (Dex) (10, 100, and 1000 pM)for 1 h prior to incubation with 0.5% CSE for 24 h. The protein expressions of Bmal1 and Clock were detected by Western blotting. Relative densities (% of control) are shown of Bmal1 and Clock in Beas-2B cells. Values are expressed as means ± SD of 3 replications. *p < 0.05 vs. control group. Full-length gels are presented in Supplementary Fig. 6.

A.Cells were treated with Bmal1-directed siRNA3 for 48 h before cells were exposed to 0.5% CSE for a further 1 h compared with siControl cells. The effects of siRNA3 knockdown of Bmal1 on 0.5% CSE-induced MEK/ERK (p-ERK) and p38 MAPK (p-p38) activation was determined after 1 h. B. Cells were treated with Clock-directed siRNA1 for 48 h before cells were exposed to 0.5% CSE for a further 1 h compared with siControl cells. The effects of siRNA1 knockdown of Clock on 0.5% CSE-induced MEK/ERK (p-ERK) and p38 MAPK (p-p38) activation was determined after 1 h. Values are expressed as means ± SD of 3 replications. *p < 0.05 and ***p < 0.001 vs. control group. #*p* < 0.05 and ##*p* < 0.001 vs. siControl group. Full-length gels are presented in Supplementary Fig. 7.

(A) Bmal1 knockdown Cells were pre-treated with MEK/ERK (PD98059, 20 µM) inhibitor, pH2AX protein expression, p16 and p21 mRNA expression were assessed. (B) Bmal1 knockdown Cells were pre-treated with p38 MAPK (SB203580, 20 µM) inhibitor, pH2AX protein expression, p16 and p21 mRNA expression were assessed. (C) Clock knockdown Cells were pre-treated with MEK/ERK (PD98059, 20 µM) inhibitor for 1 h, pH2AX protein expression, p16 and p21 mRNA expression were assessed. (D) Clock knockdown Cells were pre-treated with p38 MAPK (SB203580, 20 µM) inhibitor for 1 h, pH2AX protein expression, p16 and p21 mRNA expression were assessed. Values are expressed as means ± SD of 3 replications. ***p < 0.001 vs. control group. ###*p* < 0.001 vs. siRNA group. Full-length gels are presented in Supplementary Fig. 8.

## Supplementary Information

**Additional file 1.** 

**Additional file 2.** 

**Additional file 3.** 

**Additional file 4.** 

**Additional file 5.** 

**Additional file 6.** 

**Additional file 7.** 

**Additional file 8.** 

## Data Availability

All supporting data are included within the main article and are available by contacting the corresponding author.
