# Supplementary material for: Circadian clock gene Clock-Bmal1 regulates cellular senescence in Chronic obstructive pulmonary disease
Source: BMC Pulm Med. 2022 Nov 22;22:435. doi: 10.1186/s12890-022-02237-y (PMC9682805; doi:10.1186/s12890-022-02237-y)
Supplement: Supplementary file 1 — Additional file 1. [file 12890_2022_2237_MOESM1_ESM.docx]

Supplementary Table 2. Baseline characteristics of the study population

|  | Healthy controls | Healthy smokers | COPD |
| --- | --- | --- | --- |
| Age,yr | 62.5 ± 7.8 | 60.7 ± 11.6 | 65.5 ± 7.6 |
| No. of males/females | 30 (25/5) | 20 (18/2) | 26 (24/2) |
| Smoking status |  |  |  |
| Never smoker | 30 | 0 | 0 |
| Ex-smoker | 0 | 12 | 16 |
| Current smoker | 0 | 8 | 10 |
| BMI | 23.5 ± 3.0 | 24.0 ± 2.9 | 23.0 ± 3.1 |
| FEV1/FVC(%) | 78.3 ± 5.6 | 76.5 ± 4.0 | 60.1 ± 7.5 |
| FEV1(%) | 90.7 ± 15.4 | 88.3 ± 13.7 | 48.8 ± 20.7 |
