# Supplementary material for: Circadian clock gene Clock-Bmal1 regulates cellular senescence in Chronic obstructive pulmonary disease
Source: BMC Pulm Med. 2022 Nov 22;22:435. doi: 10.1186/s12890-022-02237-y (PMC9682805; doi:10.1186/s12890-022-02237-y)
Supplement: Supplementary file 2 — Additional file 2. [file 12890_2022_2237_MOESM2_ESM.docx]

| Gene | Forward Primer | Reverse Primer |
| --- | --- | --- |
| Bmal1 | CTGGCTAGAGTGTATACGTTTGG | GGTCACCTCAAAGCGATTTTC |
| Clock | AAAATACTCTCTACTCATCTGCTGG | ATGGCTCCTTTGGGTCTATTG |
| Per1 | CTGCTACAGGCACGTTCAAG | CTCAGGGACCAAGGCTAGTG |
| Per2 | CCCTTCCGCATGACGCCCTACCTG | GACCGCCCTTTCATCCACATCCTG |
| Cry1 | TTACACTATGCTCATGGCGAC | GTGCTCTGTCTCTGGACTTTAG |
| Cry2 | CTCTGTCTACTGGCATCTGTC | GCTTCCAGCTTGCGTTTG |
| p16 | CGGAAGGTCCCTCAGACATC | GCAGTTGTGGCCCTGTAG |
| p21 | GCAGACCAGCATGACAGATTT | GATGTAGAGCGGGCCTTTGA |
| GAPDH | GGAGCGAGATCCCTCCAAAAT | GGAGCGAGATCCCTCCAAAAT |

Supplementary Table 1. The primers for qPCR.
