# Supplementary material for: Circadian clock gene Clock-Bmal1 regulates cellular senescence in Chronic obstructive pulmonary disease
Source: BMC Pulm Med. 2022 Nov 22;22:435. doi: 10.1186/s12890-022-02237-y (PMC9682805; doi:10.1186/s12890-022-02237-y)
Supplement: Supplementary file 3 — Additional file 3. [file 12890_2022_2237_MOESM3_ESM.pdf]

Full blots images for the main figure 2

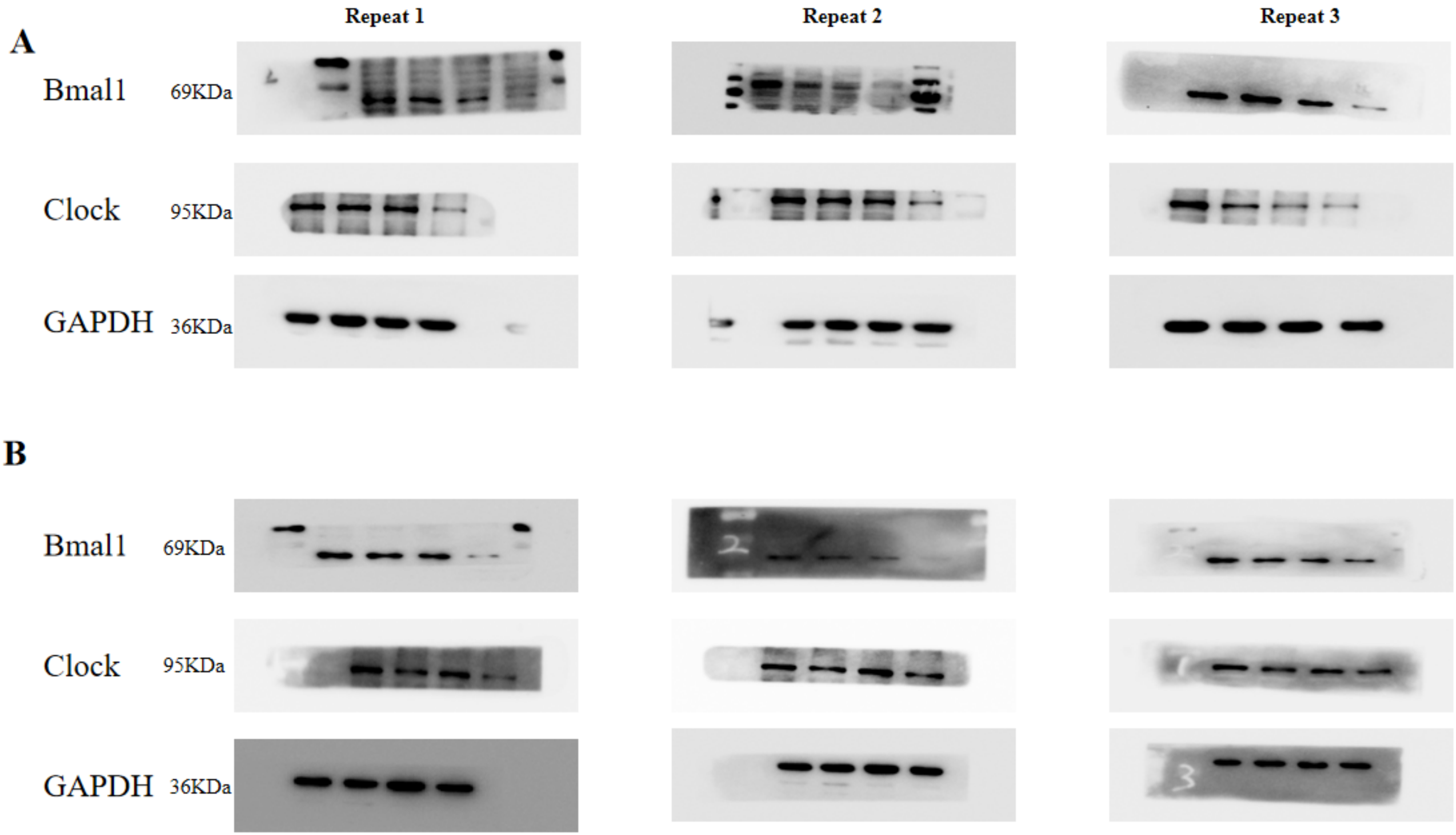

Supp. Figure 1. CSE decreased the expression of Bmal1 and Clock in Beas-2B cells. A. CSE decreased the protein levels of Bmal1 and Clock in Beas-2B cells at concentrations of CSE (0.25– 1%) for 24h. B. 0.5% CSE decreased the protein levels of Bmal1 and Clock in Beas-2B cells at times of CSE treatment (24–72 h).
