# Supplementary material for: Circadian clock gene Clock-Bmal1 regulates cellular senescence in Chronic obstructive pulmonary disease
Source: BMC Pulm Med. 2022 Nov 22;22:435. doi: 10.1186/s12890-022-02237-y (PMC9682805; doi:10.1186/s12890-022-02237-y)
Supplement: Supplementary file 4 — Additional file 4. [file 12890_2022_2237_MOESM4_ESM.pdf]

Full blots images for the main figure 3

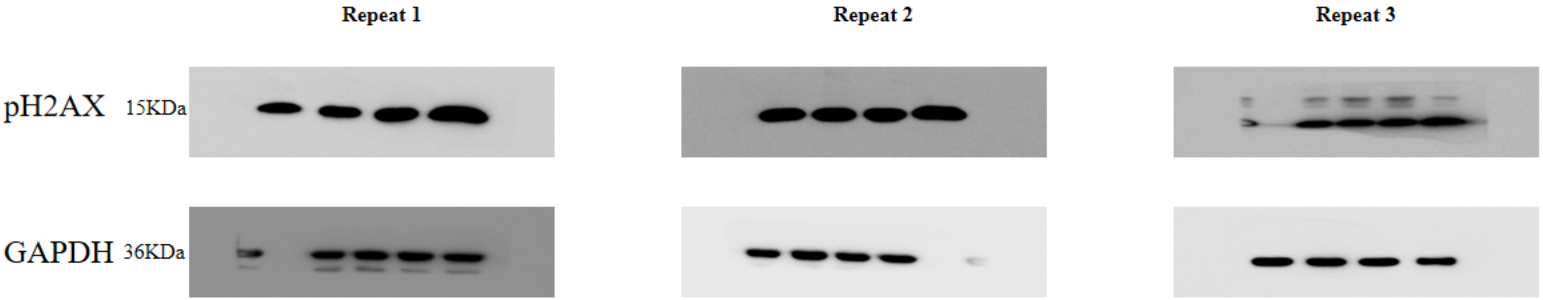

Supp. Figure 2. CSE increased cellular senescence in Beas-2B cells. CSE increased the protein levels of pH2AX in Beas-2B cells at concentrations of CSE (0.25– 1%) for 24h.
