# Supplementary material for: Circadian clock gene Clock-Bmal1 regulates cellular senescence in Chronic obstructive pulmonary disease
Source: BMC Pulm Med. 2022 Nov 22;22:435. doi: 10.1186/s12890-022-02237-y (PMC9682805; doi:10.1186/s12890-022-02237-y)
Supplement: Supplementary file 5 — Additional file 5. [file 12890_2022_2237_MOESM5_ESM.pdf]

Full blots images for the main figure 4

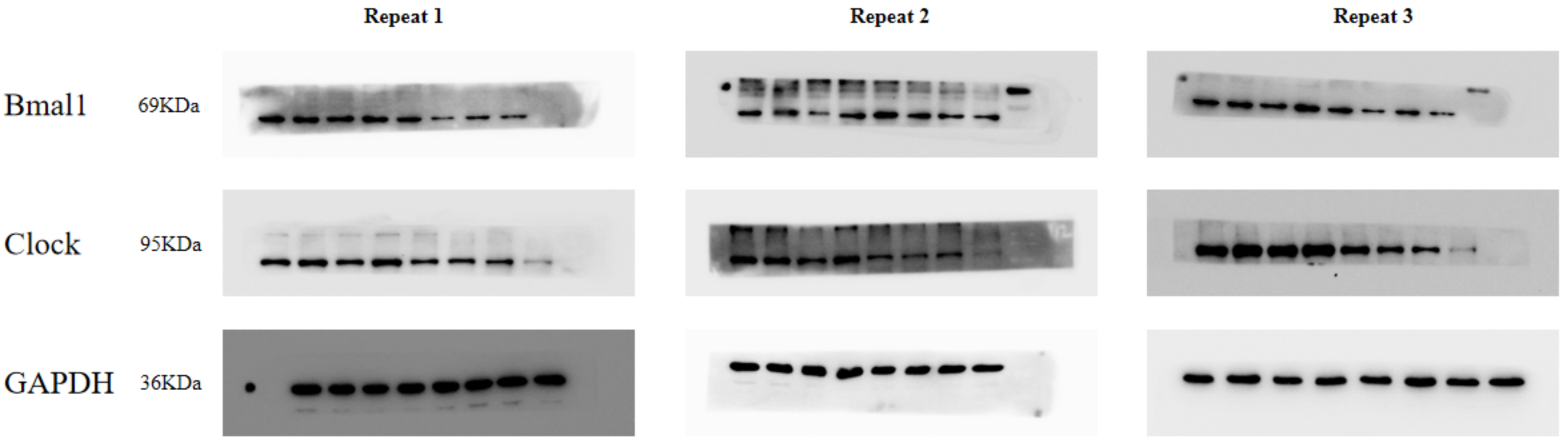

Supp. Figure 3. CSE induced circadian disturbance Beas-2B cells. Beas-2B cells were incubated with CSE (0.5%) at 6h intervals between 6h and 24h time points.
