# Supplementary material for: Circadian clock gene Clock-Bmal1 regulates cellular senescence in Chronic obstructive pulmonary disease
Source: BMC Pulm Med. 2022 Nov 22;22:435. doi: 10.1186/s12890-022-02237-y (PMC9682805; doi:10.1186/s12890-022-02237-y)
Supplement: Supplementary file 6 — Additional file 6. [file 12890_2022_2237_MOESM6_ESM.pdf]

Full blots images for the main figure 5

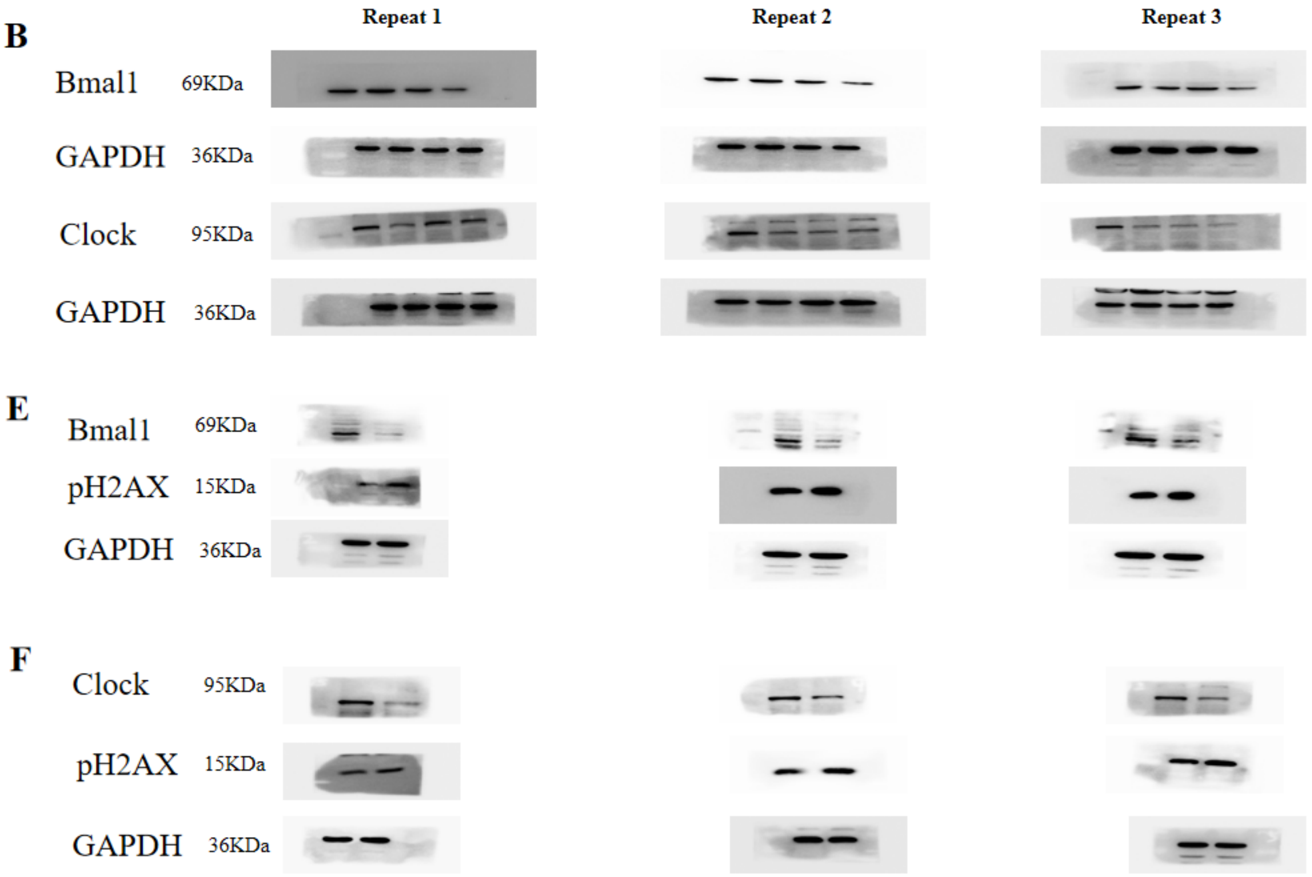

Supp. Figure 4. Knockdown of Bmal1 or Clock stimulated cellular senescence in Beas-2B cells. B.Beas-2B cells were transfected with Bmal1 and Clock siRNAs separately. E,F. pH2AX protein expression after knockdown of Bmal1 or Clock.
