# Supplementary material for: Circadian clock gene Clock-Bmal1 regulates cellular senescence in Chronic obstructive pulmonary disease
Source: BMC Pulm Med. 2022 Nov 22;22:435. doi: 10.1186/s12890-022-02237-y (PMC9682805; doi:10.1186/s12890-022-02237-y)
Supplement: Supplementary file 8 — Additional file 8. [file 12890_2022_2237_MOESM8_ESM.pdf]

Full blots images for the main figure 6

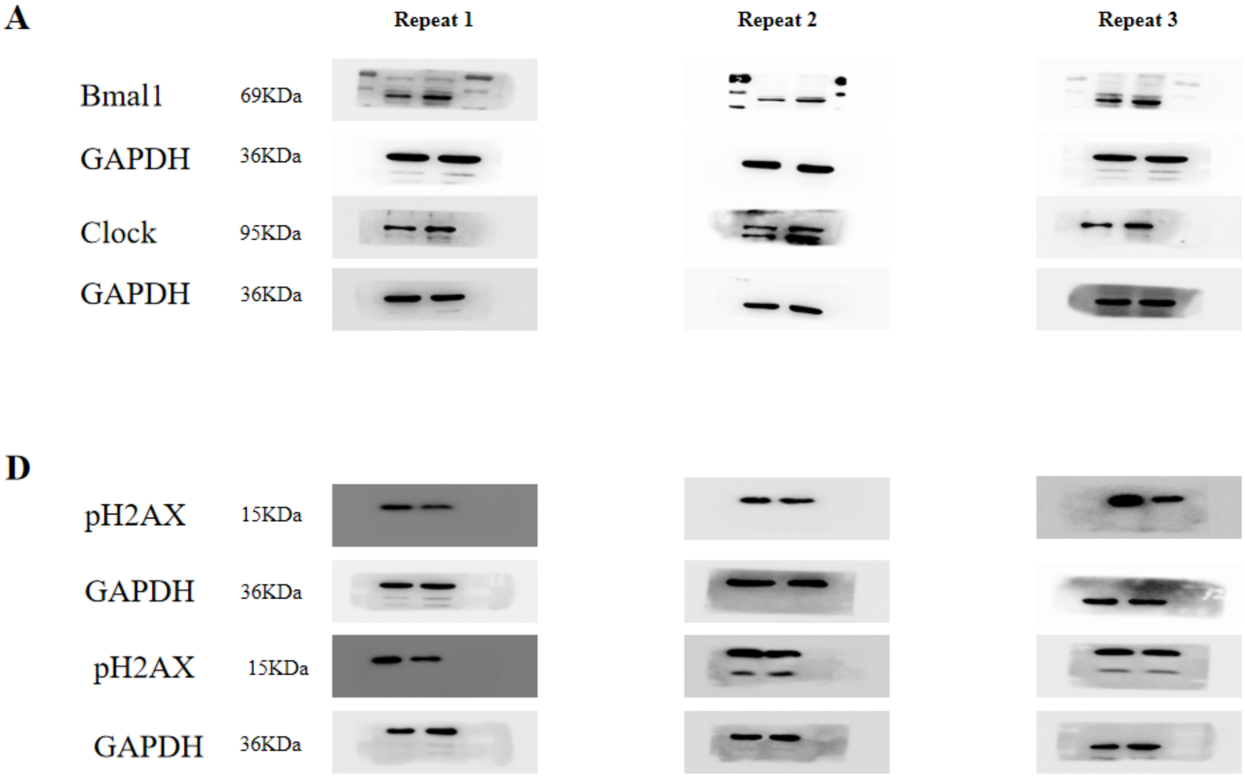

Supp. Figure 5. Overexpression of Bmal1 or Clock inhibited cellular senescence in Beas-2B cells. A.Beas-2B cells overexpressing Bmal1 or Clock were established. D. pH2AX protein expression after overexpression of Bmal1 or Clock.
